# Development and preclinical evaluation of an endonasal Raman spectroscopy probe for transsphenoidal pituitary adenoma surgery

**DOI:** 10.1117/1.JBO.30.3.035004

**Published:** 2025-03-20

**Authors:** Victor Blanquez-Yeste, Félix Janelle, Trang Tran, Katherine Ember, Guillaume Sheehy, Frédérick Dallaire, Eric Marple, Kirk Urmey, Moujahed Labidi, Frédéric Leblond

**Affiliations:** a Polytechnique Montréal, Engineering Physics Department, Montréal, Québec, Canada; b Centre de recherche du Centre hospitalier de l’Université de Montréal (CRCHUM), Montréal, Québec, Canada; c University of Montreal, Division of Neurosurgery, Department of Surgery, Montréal, Québec, Canada; d EMVision LLC, Loxahatchee, Florida, United States; e Institut du cancer de Montréal, Montréal, Québec, Canada

**Keywords:** Raman spectroscopy, pituitary adenoma, neurosurgery, tissue optics, biochemistry, machine learning

## Abstract

**Significance:**

For most patients with pituitary adenomas, surgical resection represents a viable therapeutic option, particularly in cases with endocrine symptoms or local mass effects. Diagnostic imaging, including MRI and computed tomography, is employed clinically to plan pituitary adenoma surgery. However, these methods cannot provide surgical guidance information in real time to improve resection rates and reduce risks of damage to normal tissue during tumor debulking.

**Aim:**

Here, we present the development of a handheld Raman spectroscopy system that can be seamlessly integrated with transsphenoidal surgery workflows to allow live discrimination of all normal intracranial anatomical structures, including the pituitary gland, and potentially tissue abnormalities such as adenomas.

**Approach:**

A fiber-optic probe was developed with a form factor compatible with endoscopic systems for endonasal surgeries. The instrument was evaluated in an *ex vivo* experimental protocol designed to assess its ability to distinguish normal intracranial structures. A total of 274 *in situ* spectroscopic measurements were acquired from six lamb heads, targeting key anatomical structures encountered in surgery. Support vector machine models were developed to classify tissue types based on their spectral signatures.

**Results:**

Binary classification models successfully distinguished the pituitary gland from other tissue structures with a sensitivity and a specificity of 100%. In addition, a four-class predictive model enabled >95% accuracy *in situ* discrimination of four structures of most importance during pituitary adenoma tumor resection, i.e., the pituitary gland, the sella turcica (ST) bone, the optic chiasm, and the ST dura mater.

**Conclusions:**

This work sets the stage for the clinical deployment of Raman spectroscopy as an intraoperative real-time decision support system during transsphenoidal surgery, with future work focused on clinical integration and the generalization of the approach to include the detection of tissue abnormalities, such as pituitary adenomas.

## 1 Introduction

Several tumors originate in the pituitary gland, which regulates hormonal balance and controls endocrine functions. This gland is located in the sellar region at the base of the brain. Pituitary adenomas represent ∼15% of all intracranial tumors and can have several consequences on human health, including local nerve compression, visual impairment, endocrine dysfunctions, and diseases, such as acromegaly, gigantism, or Cushing’s disease.^1^ The symptoms are contingent upon the size of the tumor and whether it secretes hormones.^1^^,^^2^ Endoscopic transsphenoidal resection surgery is a common treatment approach, during which pituitary adenomas and other tumors of the sellar region are removed through the nasal cavity and the sphenoid sinuses, using a rigid endoscope.^3^ The endoscope is used for visualization, while the patient is under general anesthesia. During the procedure, the anterior wall of the sphenoid sinus is identified and removed. Subsequently, the surgeon removes any remaining septations within the sinus and exposes the sella turcica (ST), the bony cavity that houses the pituitary gland. The bony wall of the sella (sellar floor) is meticulously removed with drills or punches.^4^ Subsequently, the dura mater is incised to expose the pituitary gland and the tumor. The tumor is then removed in pieces using microinstruments or suction devices. Great care is taken to preserve the normal pituitary tissue and to avoid damage to the surrounding structures, including the optic nerves superiorly and the carotid arteries laterally. This approach has become the standard for most pituitary adenomas and lesions confined to the sella turcica because of its precision, safety, and minimal invasiveness.^5^

The accurate identification and delineation of tumors during transsphenoidal surgery are of paramount importance, as the objective is to achieve a gross total resection of the tumor while minimizing damage to the surrounding normal structures. Achieving gross total resection safely is critical for local tumor control, as residual tumor volume significantly influences the likelihood of recurrence and endocrine control.^6^ Conversely, the resection of normal tissues may result in unintended consequences such as hormonal imbalances and diabetes insipidus.^7^ Invasion of surrounding parasellar tissues, such as the cavernous sinus and the dura mater, may also occur, further complicating complete resection.^8^ Microadenomas (<10  mm) are often more difficult to localize due to their sizes, whereas macroadenomas (>10  mm) are associated with greater risks of incomplete tumor resection.^9^^,^^10^ In approximately one-third of patients undergoing transsphenoidal surgery for non-functioning macroadenomas, an incomplete resection is inevitable.^7^ The development of surgical tools that distinguish tumor tissue from surrounding healthy pituitary tissue in real time, before surgical resection, could potentially mitigate this outcome.

Currently, preoperative magnetic resonance imaging (MRI) is the preferred imaging technique for tumor visualization and delimitation prior to surgical intervention. In addition, it enables neuronavigation during the procedure.^11^ However, MRI is occasionally unsuccessful in definitively identifying the site of microadenomas and *de novo*, persistent, or recurrent disease.^9^ Various intraoperative detection and imaging techniques are currently used, including intraoperative MRI, computed tomography, and ultrasound probes, which have demonstrated utility in detecting unexpected residual tumors and increasing the likelihood of achieving a gross total resection.^12^^–^^14^ Other promising intraoperative techniques include fluorescence-guided endoscopy and contact endoscopy, which enhances tissue vascularity imaging to aid in identifying adenomas.^15^^,^^16^ However, these techniques are not yet available in all neurosurgical centers, and although some require a significant amount of time, others are expensive or difficult to implement.^17^^,^^18^ As a result, neurosurgeons often rely on visual assessment, supported by preoperative MRI, to determine the nature of uncertain pituitary tissue during surgery.

A promising method for surgical guidance during transsphenoidal surgeries is near-infrared (NIR) Raman spectroscopy. This technique employs inelastically scattered light in biological tissues to reveal molecular information in the form of a spectrum. The vibrational modes of all molecules present within the illuminated biological sample contribute to the obtained spectroscopic information, particularly in the fingerprint region (wavenumber shifts: ∼400 to $1800\;{\mathrm{cm}}^{-1}$). This allows for tissue classification based on molecular composition and could identify normal tissue structures in real time as well as provide insight into tumoral molecular changes. Raman spectroscopy has been successfully employed for the identification of pituitary adenomas. In 2012, a group of researchers employed Raman spectroscopy to examine several brain tumor specimens obtained during surgical procedures, including pituitary adenomas.^19^ More recently, Austrian researchers utilized line scanning Raman microspectroscopy to differentiate among sub-types of pituitary adenoma biopsies, achieving high levels of accuracy.^20^^,^^21^ For Raman spectroscopy to become an intraoperative tool, it must be integrated into a fiber-optic probe and encapsulated in a specific endonasal design. A Raman probe could offer minimally invasive and real-time *in situ* tissue classification before resection, providing a faster alternative to post-resection histopathologic analysis.

This work presents the development of a handheld Raman spectroscopy probe designed for use during endonasal surgeries. The design and development of the instrument are presented as well as a preliminary evaluation of its performance in a preclinical setting. An *ex vivo* experimental protocol was developed to assess the capacity of the method to capture and differentiate *in situ* Raman spectral fingerprints of intracranial tissues within deceased lamb heads. Machine learning models were developed to assess the technique’s capacity to classify and differentiate the various types of tissues encountered during endonasal surgical procedures. This study is an initial step toward the development of a technique that will facilitate real-time identification of abnormalities and normal tissue structures during transsphenoidal surgery.

## 2 Materials and Methods

### 2.1 Handheld Fiber-Optic Probe Adapted to Transsphenoidal Surgery

**Fig. 1:**
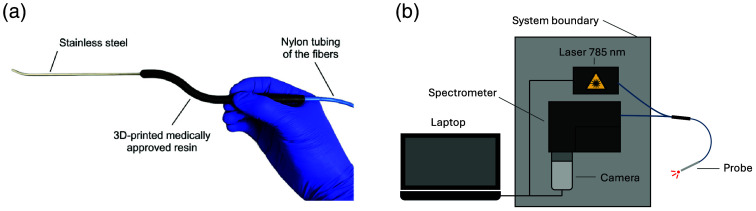
(a) Handheld endonasal Raman spectroscopy probe. Annotations indicate the different types of sterilizable materials used to fabricate the device. (b) Schematics of the complete system illustrating the enclosed module housing the near-infrared laser, the spectrometer, and the sensor, all connected to the endonasal probe. A control laptop manages all system operations.

To address the need for precise and real-time tissue identification described previously, a Raman spectroscopy probe was developed with a form factor allowing access to the pituitary gland during transsphenoidal endonasal endoscopic surgery [Fig. 1(a)]. The probe was designed with a bayoneted handle to improve access to narrow anatomical corridors and to avoid interference between the surgeon’s hands and the endoscope.^3^ The instrument includes a stainless steel extension with a length of 11 cm and a bifurcated tip at its distal end. An angled tip is a standard feature of most endoscope-assisted and microscopic surgery accessories (e.g., curettes, dissectors, hooks, and enucleators). It increases the reach of the surgical tools with a simple rotation and provides improved visualization through the endoscopic system. The length of stainless steel tubing was determined to ensure surgeons were able to reach the posterior part of the sella turcica comfortably during a procedure.

The stainless steel tubing, with an outer diameter of 2.1 mm, houses 13 multi-mode optical fibers. One central fiber, with a 300-μm core diameter, is dedicated to laser tissue illumination, whereas the remaining 12 collection fibers, each with a 200-μm core diameter, collect reemitted light through a microlens sub-assembly. The slim diameter of the tubing allows for safe and practical insertion through the sphenoid sinuses. Similar to previously published designs, interference filters were mounted within the probe tip to minimize inelastic scattering and fluorescence signal from the optical fibers material as well as to cut excitation light bleed-through to the spectrometer.^22^ The filters and lenses’ optical design ensured that measurements, with the probe in direct contact with the sample, resulted in an interrogated spot size of 500  μm in diameter. All materials used to fabricate the probe were sterilizable and biocompatible, including the wand, crafted from a 3D-printed medically approved resin.

### 2.2 Raman Spectroscopy System: Illumination and Detection Subassembly

A 3-m fiber-optic cable was used to connect the excitation fiber to an NIR laser and the collection fibers to a spectrometer [Fig. 1(b)]. The module housing the illumination and detection components was operated by a custom software responsible for all aspects of system control and data acquisition. Data processing was done using the open-source software ORPL.^23^ The complete system consisted of the handheld endonasal probe (EmVision LLC, Loxahatchee, Florida, United States), the illumination/detection module (Sentry 1000-R, Reveal Surgical Inc., Montreal, Canada), and custom software packages. The excitation fiber of the probe was connected to a continuous-wave 785-nm diode laser (class IIIB) with a maximum output power of 350 mW (Innovative Photonic Solutions, Plainsboro, New Jersey, United States). The detection components consisted of a 100-μm spectrometer slit, a diffraction transmission holographic grating, and a charge-coupled device (CCD) sensor (Newton model, Andor Technology, Belfast, United Kingdom) that was pre-cooled at −70°C. Each spectrum acquired with the system covered a range of spectral shifts from 400 to $2000\;{\mathrm{cm}}^{-1}$, with a spectral resolution of $∼8.7\;{\mathrm{cm}}^{-1}$.

The axial resolution of the system, here perhaps more accurately referred to as tissue depth sampling, varies depending on the intrinsic optical properties of the interrogated tissue. Monte Carlo light transport simulations showed that depth sampling varied from 10 to 600  μm, for absorption coefficients ($\mu_{a}$) of 0.001 to $1.4\;{\mathrm{mm}}^{-1}$ and reduced scattering coefficients ($\mu_{s}^{\prime }$) of 0.5 to $30\;{\mathrm{mm}}^{-1}$.^24^ These values cover the ranges expected for biological tissues, including intracranial structures such as the brain, bone, mucosa, and meninges.^25^^–^^27^

### 2.3 Single-Point Spectroscopic Data Acquisition Protocol

A calibration procedure was implemented on each Raman spectroscopy measurement acquired as part of the study.^23^ System calibration used a National Institute of Standards and Technology (NIST) Raman standard (SRM 2214) measurement performed prior to each tissue measurement session to account for the instrument response. A measurement on acetaminophen powder, used as a reference spectrum, was also performed to calibrate the x-axis, ensuring each camera pixel index corresponded to a spectral bin reported in inverse centimeters (${\mathrm{cm}}^{-1}$). The probe tip was cleaned with alcohol after each measurement to minimize the likelihood of contamination from tissue deposits from previous measurements.

**Fig. 2:**
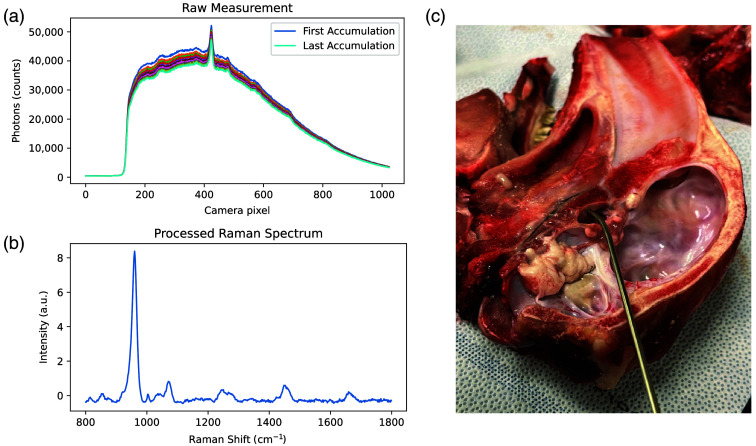
(a) Raw spectroscopic measurement using the near-infrared Raman spectroscopy probe. Repeat measurements were made for each tissue location and averaged to maximize the overall signal-to-noise ratio. A representative measurement is shown for the sella turcica bone. (b) SNV-normalized processed Raman spectrum. (c) Close-up of the endonasal probe positioned at an angle on the sella turcica bone, after removal of the pituitary gland and overlying dura mater.

Two individuals were involved with the acquisition of Raman measurements. An engineer (VBY) operated the system, whereas a neurosurgery resident (FJ) identified targeted tissue locations based on knowledge of intracranial anatomy. The neurosurgery resident held the probe in contact with the tissue during each measurement [Fig. 2(c)]. The latter was performed at fixed on-tissue laser power (40 mW) with a variable exposure time, automatically adapted to maximize the photonic count while avoiding CCD sensor saturation.^28^ Each spectral measurement consisted of averaging 10 spectral accumulations, with total acquisition times ranging from 3 to 11 s, depending on the tissue type. Brain tissues, such as white and gray matter, required longer acquisition times—8.9 and 10.1 s on average, respectively—due to their lower signal intensities. All measurements were carried out under low ambient light to minimize background contributions and allow inelastically scattered photon signals to capture the largest possible fraction of the camera’s dynamic range.

### 2.4 *In Situ* Raman Spectroscopy of Normal Intracranial Structures

A pre-clinical *ex vivo* study protocol was designed and implemented to assess the ability of the Raman spectroscopy probe to detect macroscopically distinguishable intracranial structures, including the brain. A total of six lamb heads were purchased at a butcher shop. The heads were all cut in half with a bandsaw through a sagittal plane, exposing the different tissues to be probed within 48 h of the animals’ death. The half-heads were rinsed with distilled water to remove bone residue without affecting tissue integrity.

**Fig. 3:**
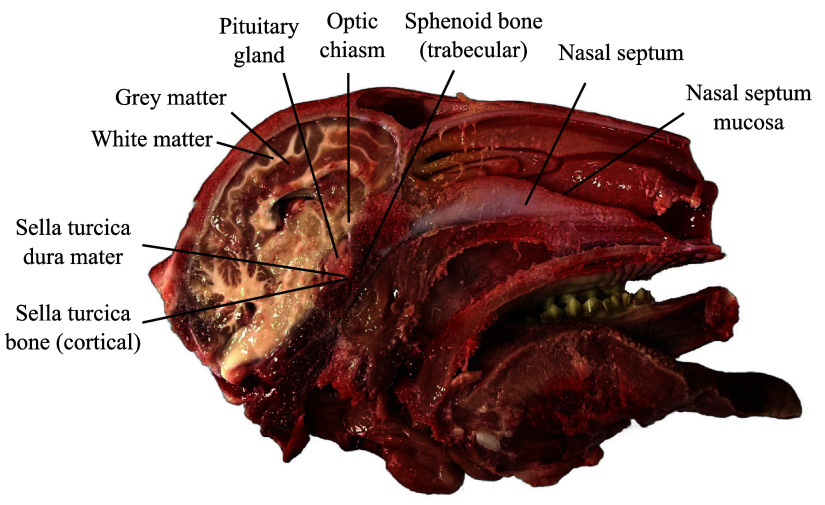
Annotated image of a lamb half-head, displaying the various tissues measured using a Raman spectroscopy probe in the scope of the study.

*In situ* spectral measurements were acquired for nine different anatomical structures: the pituitary gland, the optic chiasm, the sella turcica bone, the dura mater of the sella turcica, the sphenoid bone, the nasal septum, the mucosa covering the nasal septum, and the white and gray matter in the brain (Fig. 3). Anatomical regions encountered during transsphenoidal pituitary adenoma resection are the pituitary gland, the optic chiasm, the sella turcica bone and dura mater, and the sphenoid bone. The nasal mucosa and nasal septum are also commonly encountered during the approach to the region where tumor debulking is done. Although not relevant to pituitary adenoma resection, white and gray matters were considered here nevertheless for reference, as multiple publications reported Raman spectra of the brain,^29^^,^^30^ including past work from our group.^31^^,^^32^ In total, between 15 and 59 spectra were acquired for each of the nine tissue categories across six different lamb heads. Visual classification of the tissues and selection of the measurement sites was performed by the neurosurgery resident.

Measurements were conducted on areas accessible with the probe’s tip within each tissue type. Specifically for bone tissues, samples were collected from the cortical region of the sella turcica bone while probing the sphenoid bone focused on its trabecular area, exposed by the cut through the sagittal plane. The trabecular, or spongy region, is the bone’s porous lattice structure fully enclosed by opaque cortical bone.

Some tissues were measured after the removal of other structures that initially obstructed the probe’s access. For instance, to obtain spectra from the sella turcica bone, the pituitary gland and the dura mater covering the bone were carefully resected [Fig. 2(c)]. For the dura mater, the *in situ* measurements focused on the part of the membrane overlying the sella turcica bone, known as the sella turcica dura, before its resection. The bone was directly beneath the dura, aligned with the probe’s field of view. In humans, the dura in this region typically measures ∼0.4  mm in thickness^33^^,^^34^ and is expected to be even thinner in juvenile sheep. The comparison of this value with the probe’s estimated sampling depth highlights a potential contribution of Raman scattering originating from the underlying sella turcica bone in the spectral acquisitions corresponding to the sella turcica dura.

### 2.5 Pituitary Gland Measurements Under Different Experimental Conditions

Three different tissue preparation methods were tested to acquire Raman spectra of the pituitary gland, aiming to evaluate spectral consistency and classification model robustness under different experimental conditions. First, *in situ* measurements were made prior to excision, as described in Sec. 2.4, directly on the glands. Then, *ex situ* measurements were made on the excised glands. Finally, the glands were sectioned into four or five slices of approximately equal thickness (∼2  mm) along the axial plane, resembling the specimens acquired during surgery. These measurements will be herein referred to as “in-section” acquisitions. Three out of six lamb heads were selected for this experiment. The resulting dataset included 29 *in situ* measurements, 21 *ex situ* measurements, and 27 in-section measurements. All acquisitions were completed within an hour to minimize the impact of tissue degradation.

### 2.6 Spectroscopic Data Processing and Analysis

#### 2.6.1 Spectroscopic data pre-processing

The following data processing steps were applied to each raw spectroscopic measurement [Fig. 2(a)] using the ORPL open-source software:^23^ (1) subtraction of a dark count background measurement acquired with the laser turned off prior to each measurement (e.g., to remove signal from residual ambient light sources); (2) removal of cosmic ray events; (3) x-axis calibration using the known positions of Raman peaks and instrument response correction from spectral measurements acquired in calibration materials (acetaminophen, NIST 785 nm Raman); (4) averaging of successive measurements (i.e., accumulations) acquired at the same location; (5) baseline subtraction using a custom algorithm, BubbleFill, with the minimum “bubble” parameter of $50\;{\mathrm{cm}}^{-1}$; and (6) curve smoothing using a Savitzky–Golay filter of order 3 with a window size of 1. The spectra were then truncated to keep only the spectral bins between 800 and $1800\;{\mathrm{cm}}^{-1}$, followed by standard–normal–variate (SNV) normalization [Fig. 2(b)]. As a result of the SNV normalization, the “intensity” associated with each spectral bin must be interpreted as a deviation relative to the average of all detected inelastic scattering contributions across the spectral domain.

#### 2.6.2 Spectral quality factor to assess measurement reliability

A spectral quality factor (QF) metric was computed for each SNV-normalized spectral fingerprint to assess the quality of the Raman signals. This metric measured the average signed squared intensity, quantifying the likelihood that the signal is associated with a random probability distribution.^23^ If a signal contains few large and narrow peaks, the QF will be large (close to 1), whereas if a signal contains many small and broad peaks, the QF will be smaller. Finally, a signal that contains only stochastic noise will have a QF of 0. The quality factor is defined as

$$\mathrm{QF}≔\frac{1}{N}\overset{N}{\underset{i=1}{\sum }}\;\mathrm{sgn}(r_{i})\cdot r_{i}^{2},$$

where r is an SNV-normalized Raman spectrum, and sgn(x) returns the sign (+1 or −1) of the variable x. To ensure high-quality Raman signals for subsequent analyses and machine learning models, a cutoff value of QF was established. The optimal threshold value of 0.3 was chosen to maximize models’ performances while avoiding class imbalances and was consistent with previous work.^28^^,^^32^ This led to the elimination of 10 out of the 284 spectra collected in this study.

#### 2.6.3 Spectral angle mapper to assess the similarity of different measurements

The spectral angle mapper (SAM) was used as a quantitative measure of the similarity between two spectra. The SAM is a metric that measures the angles among spectra in a multi-dimensional space, where each spectral bin is a dimension.^35^ It is defined as

$$\theta =\cos^{-1}\;\frac{\sum_{i=1}^{n}X_{i}Y_{i}}{\sum_{i=1}^{n}X_{i}^{2}\cdot \sum_{i=1}^{n}Y_{i}^{2}},$$

where $X_{i}$ and $Y_{i}$ are the i’th bins (wavenumber shift values) associated with spectra X and Y represented as n-dimensional vectors. Identical vectors will have a SAM value of θ=0  deg, whereas θ=90  deg corresponds to orthogonal vectors (i.e., unrelated in spectral shape).

#### 2.6.4 Band assignment for all interrogated tissue types

The relatively narrow Raman peaks ($<50\;{\mathrm{cm}}^{-1}$), obtained after baseline signal removal, can be associated with their corresponding vibrational modes and biomolecular assignments. However, in biological samples, identifying the specific biomolecular compound giving rise to a Raman peak is complicated by the great number of molecular bonds and their complex structures present in biological tissues. Recent Raman spectroscopy studies have significantly contributed to facilitating identification by compiling published Raman spectral interpretations of biological tissues to develop databases.^36^ The peak assignments of the spectral fingerprint acquired in this study were based on multiple studies focused on Raman spectroscopy of pure molecules, such as proteins, lipids, and amino acids.^37^^–^^39^ All the assignments were then correlated with databases and cross-checked with their original publications and samples.

### 2.7 Machine Learning Tissue Classification Models

Predictive modeling approaches were used to assess the ability of Raman spectroscopy to distinguish different types of intracranial tissues. Two machine learning classification modeling schemes were used to train, validate, and test models with the *in situ* Raman spectra. Binary models were initially developed to assess the ability of the technique to distinguish the pituitary gland from all other normal structures. Then, a more clinically relevant model was developed in the form of a four-class model designed to discriminate four structures of importance for surgical guidance during pituitary adenoma tumor resection.

The first class of models (model I) consisted of multiple two-class classifiers, each specifically designed to differentiate the pituitary gland spectra from those of a single other probed tissue. In addition, one model was trained to distinguish the gland from all the other tissues combined. This approach focused on distinguishing normal (healthy) gland tissue from molecularly distinct tissues, particularly surrounding tissues, such as the dura mater and bone of the sella. This served as a foundational step toward differentiating between normal gland tissues and adenomas in human patients. No classifier was built to distinguish the gland from the nasal mucosa and septum measurements, as the number of spectra collected for these tissues (n=15 each) was deemed insufficient to provide a robust testing set. In addition, these distinctions were considered less clinically relevant. The second class of models (model II) was a multi-class model developed to differentiate four neighboring tissues commonly encountered during surgery: the pituitary gland, the sella turcica bone, the optic chiasm, and the sella turcica dura mater. This model highlighted the potential of the probe for surgical guidance by providing real-time tissue mapping to improve procedural safety during adenoma resection.

All models were tested on hold-out datasets that were not used during training and validation. For all tissues, between 33% and 40% of the measurements were reserved for testing, whereas the remaining 60% to 67% were used for training and validation. To ensure the ability to classify tissues from new and independent individuals could be tested, the training and testing datasets were completely separated by heads, ensuring that no data from the same individual were used in both training/validation and testing.

The methodology used to develop the models was described elsewhere.^32^ Briefly, each processed Raman spectrum consisted of ∼1000 spectral bins, ranging from 800 to $1800\;{\mathrm{cm}}^{-1}$. Prior selection of Raman peaks was applied to prevent the inclusion of features that corresponded to stochastic noise rather than actual Raman signal.^40^ Then, the number of spectral bins used for machine learning modeling was reduced to N<20 spectral features through a linear support vector machine (SVM) approach with L1-regularization (Lasso regression) optimization, which promotes sparsity by selecting only the most relevant spectral features. The optimal N was determined via cross-validation to balance model performance and prevent overfitting. The selected features corresponded to the spectral bins with the largest absolute coefficients from the linear SVM. These features were used to train and validate the final classification SVM model with L2-regularization (Ridge regression). Hyperparameter optimization was applied to the regularization methods of both the feature selection and classification phases to maximize predictive accuracy using fivefold cross-validation. Monitoring accuracy across the folds minimized the risk of overfitting. The cross-validation results were summarized in confusion matrices by aggregating the results from each fold.

Once the optimal parameters were selected for all models, performance was assessed using the hold-out testing datasets, with accuracy metrics, confusion matrices, and receiver operating characteristic (ROC) analysis for the two-class model I. The multi-class model II results on the validation and testing data were summarized in a four-by-four confusion matrix. Subsequently, *ex situ* and in-section pituitary gland measurements were classified using model II, which had been trained solely on *in situ* measurements, to assess the ability to classify spectra independent of the acquisition method. These results were also presented in four-by-four confusion matrices.

## 3 Results

### 3.1 *In Situ* Tissues Mean Spectral Fingerprint and Band Assignment

**Fig. 4:**
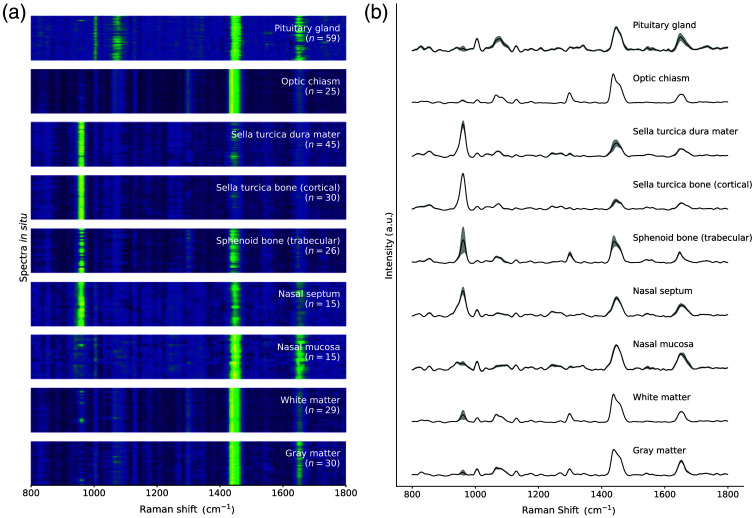
(a) Spectrogram showing the entire *in situ* Raman spectroscopy dataset. (b) Average spectra per tissue, with the corresponding inter-measurement variance shown for each spectral bin.

All the SNV-normalized spectra collected *in situ* for each type of tissue were represented in the form of spectrograms [Fig. 4(a)]. In those spectrograms, each line corresponded to an individual spectrum depicted as a yellow (maximum value) to blue (minimum value) gradient. The name and total number of processed spectra for each tissue were shown on the spectrogram for each tissue category. The average Raman spectrum for each tissue type was also drawn along with the variance across all measurements [Fig. 4(b)]. The variance was computed for each spectral bin and was represented as a semi-transparent gray band.

The assignments of the spectral bands composing the mean spectral fingerprints of the tissues are summarized in Table 1. For each visually identifiable spectral peak, a biomolecular association with the corresponding vibrational modes was attempted. The relative intensity (across all tissues) of the peaks was categorized using a scale from 1 to 5 and represented with asterisks ranging from the lowest contribution (*) to the highest (*****).

**Table 1:** Principle vibrational modes, band assignments, and corresponding relative concentration in each tissue type indicated by the number of asterisks from lowest (*) to highest (*****).

Peak center (${\mathrm{cm}}^{-1}$)	Main vibrational modes	Biomolecular assignment	Pituitary gland	Optic chiasm	Sella turcica bone and dura mater	Sphenoid bone	Nasal septum and mucosa	Cerebral matter
822 to 825	C–C stretching	Proteins (tyrosine)	*	—	—	—	*	—
856	C–C vibration	Proteins (tyrosine)	*	—	*	*	*	—
940	ν(C–C) skeletal	Proteins (N–C–C)	*	—	—	—	**	—
961	Calcium phosphate stretch band	Calcium hydroxyapatite, cholesterol, and protein (tryptophan and valine)	*	*	Bone: ***** Dura: ****	****	Bone: **** Mucosa: **	*
1004	Symmetric stretching and ring breathing modes	Protein (phenylalanine and tryptophan)^37^^,^^39^	**	*	*	—	**	*
1064	Skeletal C–C stretch	Lipids and triacylglycerols (C–C)	**	**	—	*	—	*
1078	C–C and C–O stretching mode	Phosphate (dihydrogen phosphate and hydrogen phosphate) and lipid (C–C)^38^	**	*	*	*	—	*
1128	C–N stretching	Fatty acid (C–C), protein (C–N and serine),^37^^,^^39^ and glucose	*	*	—	*	*	**
1298	CH_2_ twisting and deformation	Lipid (CH_2_)	*	**	—	**	—	White: ** Gray: *
1437 to 1439	CH_2_ deformation, scissoring, and bending	Lipid (CH_2_ and CH_3_)^38^ and protein (C–H, isoleucine, and valine)^37^^,^^39^	***	****	Bone: * Dura: **	***	Bone: ** Mucosa: ***	****
1446 to 1452	CH_2_/CH_3_ deformation	Protein (histidine, leucine, lysine, methionine, serine, and threonine)^37^^,^^39^	****	***	Bone: * Dura: **	***	Bone: *** Mucosa: ****	***
1649 to 1652	C=O and C=C stretching and α-helix	Lipid (unsaturated C=C)^38^ and protein (amide I)^37^^,^^39^	***	**	*	**	Bone: ** Mucosa: ***	White: ** Gray: ***

The peak at $961\;{\mathrm{cm}}^{-1}$, often referred to as the mineral peak,^36^ is associated with calcium hydroxyapatite, a mineral component that makes up ∼60% of bones.^41^ It is highly present in bone spectra, such as the sella turcica, sphenoid, and nasal septum bone. The peak at $1004\;{\mathrm{cm}}^{-1}$ can be attributed to proteins, such as phenylalanine and tryptophan.^37^^,^^39^

Both 1064- and $1298\text{-}{\mathrm{cm}}^{-1}$ peaks are primarily due to lipids, with a higher contribution in the pituitary gland, optic chiasm, and cerebral matters. Lipids can be found in the form of phospholipids, cholesterol, and triglycerides in the pituitary gland.^42^ The optic chiasm and cerebral matter are high in lipids, which compose 70% to 80% of myeline,^43^^,^^44^ the insulating layer surrounding nerve fibers. Those peaks are also present in sphenoid bone, likely because measurements were taken from the spongy, trabecular region of the bone, containing bone marrow. Marrow is composed of hematopoietic cells (bone stem cells), and marrow adipose tissue is high in lipid and supportive stromal cells.^45^ It is also associated with a lower mineral density than cortical bones,^45^ correlating with the lower intensity of the mineral peak (at $961\;{\mathrm{cm}}^{-1}$) in the spectral fingerprint of the trabecular sphenoid bone. Those spectra also show significantly more variance than those of the cortical region of the sella turcica bone. This variability reflects the trabecular bone’s porous lattice structure, which contains more connective tissues, marrow, fat, and blood vessels, contributing to its greater heterogeneity compared with the denser, more uniform cortical bone.^46^ Only 20% of the volume of the trabecular compartment is bone.^46^

The peak with the highest contribution in the components of the central nervous system (CNS), such as both cerebral matters and the optic chiasm, is at 1437 to $1439\;{\mathrm{cm}}^{-1}$. It has been associated with proteins, lipids, and DNA/RNA. The aggregation of these different molecular species represents the distribution of proteins and genetic information in nerve fibers of the CNS. Similarly to lipids, different proteins can be found in myelin, such as myelin basic protein, proteolipid protein, isoleucine, and valine.^44^ This peak also contributes significantly to the spectra of the pituitary gland and the nasal mucosa. However, the highest contribution for both tissues is the peak at 1446 to $1452\;{\mathrm{cm}}^{-1}$, which encompasses fatty acids and other lipids. These lipids are important components of the pituitary glands’ lipid composition.^42^ The peak at 1649 to $1652\;{\mathrm{cm}}^{-1}$, associated with a mix of lipid and protein contribution, such as amide I, is present in most tissues. Its lowest relative contribution appears in the cortical bone’s spectrum, similar to the other peaks associated with proteins and lipids.

**Fig. 5:**
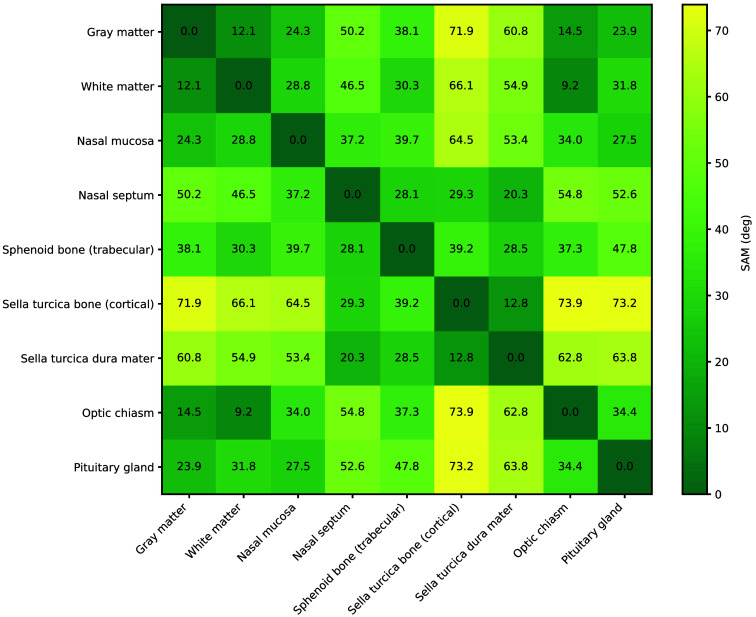
SAM computed among the average Raman spectra for all possible combinations of tissue types. SAM values range from 0 deg for identical spectral fingerprints to a maximum of 90 deg when the spectra are highly dissimilar.

The similarity among the average spectral fingerprints of the tissues was assessed by computing the SAM of all possible pairs of average spectral fingerprints (Fig. 5), which was represented by an average SAM value of 41.1 deg. The average spectra of the optic chiasm and white matter yielded the lowest SAM value of 9.2 deg, probably due to their similar composition of myelinated axons, which gives them their white appearance. The angles computed for these tissues when compared with gray matter were 12.1 deg for white matter and 14.5 deg for the optic chiasm. Although all three tissues share similar compositions as integral components of the CNS, gray matter differs as it is primarily composed of unmyelinated axons, neuronal cell bodies, and dendrites.^47^

The visual resemblance between the spectral fingerprints of the dura mater and the bone of the sella turcica is corroborated by a SAM value of 12.8 deg among their mean spectra. This similarity is notable given the distinct compositions of these tissues: the dura mater is a membrane composed primarily of connective tissue rich in collagen and elastic fibers,^34^ whereas the underlying tissue consists of dense cortical bone. The presence of a varying and pronounced mineral peak at $961\;{\mathrm{cm}}^{-1}$, characteristic of bone tissues, is notable, though less intense than in the sella turcica bone’s fingerprint. This peak’s intensity, along with the varying higher intensities of peaks associated with proteins and lipids in the dura mater’s mean spectrum compared with the bone, supports the hypothesis of an underlying bone contribution within the dura mater’s Raman signals. All the other SAM measurements among the average spectra of different tissues were superior to 20 deg, indicating less similarity.

Although SAM provides a useful dataset overview by comparing the average spectra, its effectiveness in classifying individual spectroscopic measurements is limited. As SAM relies on spectral angle calculations, baseline variations and noise can shift vector orientation without altering key spectral features, resulting in misleading similarity measures. For all tissues, some comparisons of spectra within that same tissue yielded higher SAM values than those with spectra from different tissues (Supplemental Material Fig. S1), emphasizing this limitation. In contrast, machine learning models provide superior accuracy, robustness to noise, and scalability, making them more suitable for clinical applications. Unlike SAM, which depends on a predefined reference and is greatly affected by spectral variability, SVM can generalize to new spectra, select key spectral features, and mitigate overfitting through regularization.

### 3.2 Binary Tissue Classification Models

**Fig. 6:**
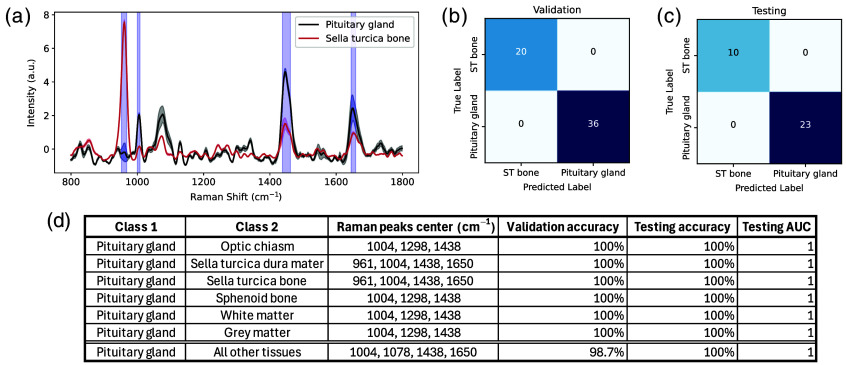
Results of the two-class predictive models classifying the pituitary gland and the ST bone. (a) Average Raman spectra with the bands from which features were used for modeling highlighted in blue, confusion matrices representing the classification results associated with the (b) cross-validation and (c) testing phases of model development. (d) Table summarizing the results of all two-class models, including the Raman bands from which features were used by the models, validation and testing predictive accuracies as well as the AUC of the testing set ROC analysis curve.

Visual inspection of the average Raman spectra for each tissue type revealed distinct spectral bands enabling discrimination, which was further confirmed by the SVM models. The Raman peaks from which spectral features were selected for machine learning modeling in distinguishing the pituitary gland from the sella turcica bone were highlighted in graphs showing average spectra for these two tissue types [Fig. 6(a)]. Key peaks used to separate the gland from the bone included the peaks centered at 961, 1004, 1438, and $1650\;{\mathrm{cm}}^{-1}$, attributed to mineral, phenylalanine (proteins), lipids, and proteins (amide I) contributions, respectively. The model was able to predict tissue class with 100% sensitivity and 100% specificity, both during the cross-validation phase [Fig. 6(b)] and when it was directly applied to an independent testing set [Fig. 6(c)].

For conciseness, spectral comparison graphs and confusion matrices were not shown for the models developed to distinguish the pituitary gland from the optic chiasm, the dura mater, the sphenoid bone, and the white and gray matters. However, the performance metrics, as well as the main peaks from which spectral features were extracted for modeling, were tabulated [Fig. 6(d)]. On average, 12 spectral features were used by the models which all led to 100% validation and testing accuracies. All models were associated with an ROC curve area under the curve (AUC) of 1. Discrimination from the sella dura mater required features from the same peaks as the bone, i.e., 961, 1004, 1438, and $1650\;{\mathrm{cm}}^{-1}$. Discriminating the pituitary gland from the other structures (optic chiasm, sphenoid bone, and white and gray matters) was possible with identical spectral peaks. The classification was done by selecting features from the bands centered around 1004, 1298, and $1438\;{\mathrm{cm}}^{-1}$.

Lastly, the model designed to differentiate the pituitary gland from all other probed tissues used 20 spectral bins and misclassified 1 out of 36 pituitary gland spectra and 1 out of 115 spectra from other tissues during cross-validation, achieving a sensitivity of 97.2% and a specificity of 99.1%. In the hold-out test set, all spectra were correctly classified, resulting in a testing accuracy of 100%, which was tabulated along with the other performances [Fig. 6(d)].

### 3.3 Multi-Class Tissue Classification Model

**Fig. 7:**
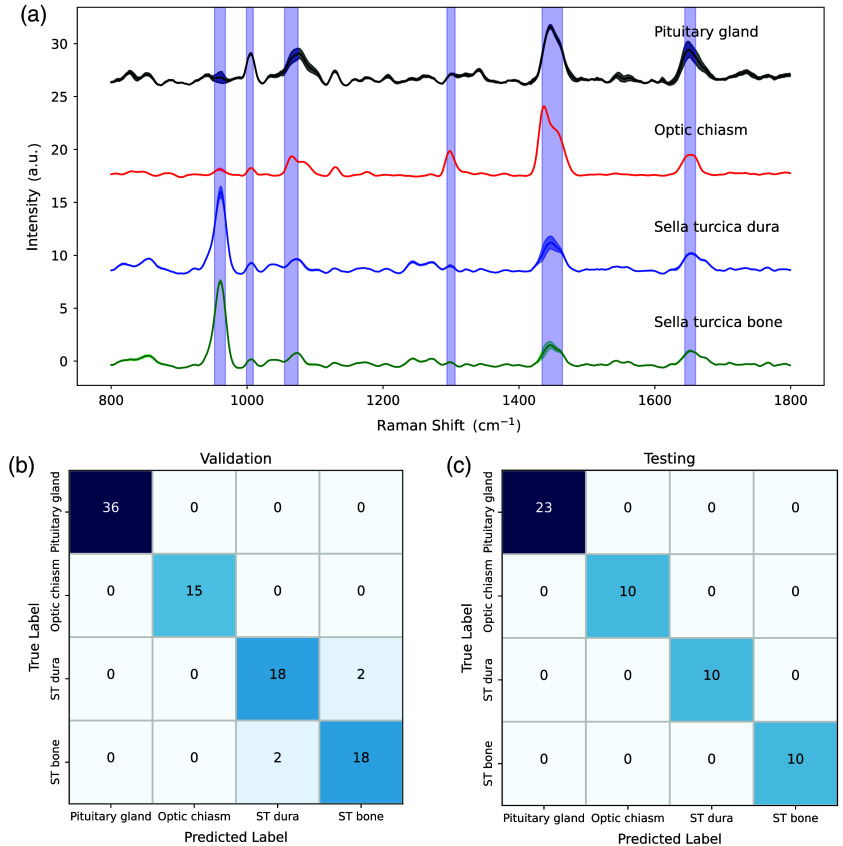
Results of the multi-class predictive model (model II). (a) Average Raman spectra of the tissue measurements used to develop a machine learning model. The bands from which features were used for modeling are highlighted in blue. (b) Confusion matrix representing the classification results associated with the cross-validation phase of model development. (c) Confusion matrix showing the results of the machine learning model on the testing hold-out dataset.

A multi-class predictive model was then developed that could be used as a real-time decision support system for neurosurgical guidance. The model was trained, validated, and tested using the *in situ* spectral datasets associated with the pituitary gland, the optic chiasm, the sella turcica dura, and the sella turcica bone [Fig. 7(a)]. The spectral peaks chosen to perform multi-class discrimination were highlighted in blue on the spectral graphs and correspond to the bands centered around 961, 1004, 1064, 1298, 1438, and $1650\;{\mathrm{cm}}^{-1}$. A total of 19 spectral bins (i.e., intensities corresponding to different wavenumbers) were used as features for the model. The model resulted in an accuracy of over 95% in training/validation, with only dura mater and bone spectra being misclassified, with validation accuracies of 90% for both classes. The model achieved an accuracy of 100% when applied to the hold-out test set. The confusion matrices were shown for the validation [Fig. 7(b)] and testing [Fig. 7(c)] phases. The model misclassified 4 spectra out of 94 in the training/validation while correctly classifying all the testing spectra that were not used for the training and tuning of the model.

### 3.4 Comparison of *In Situ*, *Ex Situ*, and Sectioned Pituitary Gland Raman Acquisitions

**Fig. 8:**
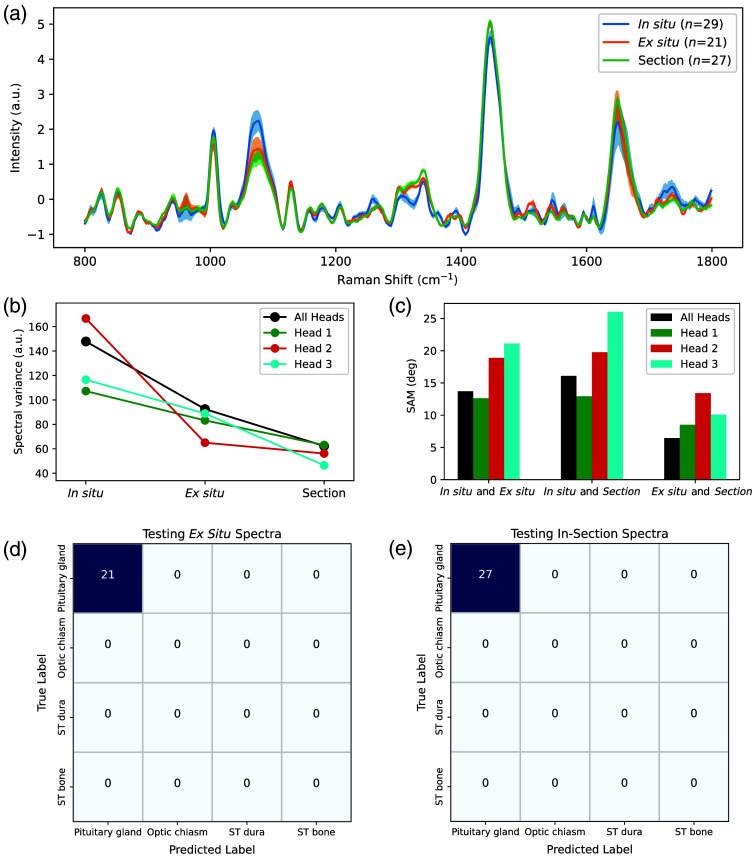
(a) Average Raman spectra of the pituitary glands associated with each tissue preparation method (*in situ*, *ex situ*, in-section) with the corresponding inter-measurement variance shown. (b) Variance summed over all spectral bins for each acquisition method and (c) SAM values computed among the average spectra from all combinations of tissue preparation methods. Variances and SAM values are represented for the spectra averaged for each individual lamb head as well as for the spectra averaged across all three lamb heads. Confusion matrices representing the classification results obtained when applying multi-class model II to measurements associated with (d) *ex situ* and (e) in-section tissue preparation methods.

The average Raman spectral fingerprints of the pituitary gland for all three different acquisition methods (*in situ*, *ex situ*, and in-section) were represented with the corresponding variance associated with all spectral bins [Fig. 8(a)]. Those fingerprints seemed visually similar while sharing some peak intensity differences, notably at 1064, 1341, and $1650\;{\mathrm{cm}}^{-1}$. The total variance of the spectra for each measurement method, represented in Fig. 8(b), was calculated as the sum over all spectral bins. *In situ* measurements exhibited the highest total variance, followed by *ex situ* measurements, whereas the measurements from sectioned glands showed the lowest total variance. This trend was consistent across all individual heads, as illustrated in the figure. The assessment of signal similarity among different methods, based on SAM measurements among the average spectra [Fig. 8(c)], showed that *in situ* and *ex situ* spectra shared greater spectral similarity compared with *in situ* and in-section spectra. Meanwhile, *ex situ* and in-section spectra exhibited the lowest SAM value, indicating the highest similarity. This relative ranking of similarity remains consistent across all individual heads.

Multi-class model II, which was trained using *in situ* measurements, accurately classified all the *ex situ* and in-section spectra as pituitary gland tissue, illustrated in the testing confusion matrix of both methods (Fig. 8). This demonstrates our ability to classify the glandular tissue with perfect accuracy, regardless of the acquisition method.

## 4 Discussion and Conclusion

Raman spectral fingerprints of different intracranial tissues critical to transsphenoidal surgery were measured and subsequently analyzed with biomolecular assignments and similarity comparisons. The alignment of peak intensities with each tissue’s biomolecular composition substantiated the accuracy and reliability of the Raman signal acquisition process. The results of both binary and multi-class models demonstrated the ability of the technique to classify structures *in situ* with the Raman probe.

The SAM metric was used to assess the similarity of average spectral fingerprints of the different tissues. It allowed a better comprehension of the obtained spectra and validation with previous studies. This metric could be further explored to regroup similar tissues prior to machine learning classification. The observed similarity between the spectral fingerprints of the dura mater and the bone of the sella turcica is likely caused by the spectral contribution of the underlying bone in the spectrum taken on the thin dura mater, as suspected prior to the acquisitions. Nevertheless, multi-class model II was able to accurately classify those tissues. In the context of a clinical study, dura mater tissues in adult human patients are expected to be thicker than in lamb, potentially minimizing that effect. However, these findings suggest that the impact of the tissue-dependent sensing depth needs to be monitored.

Classifying pituitary adenomas from normal glands in human patients is expected to be more challenging than the classifications done by model I, as adenomas share a more similar molecular structure with normal glands, leading to more closely related spectral fingerprints, than normal glands and surrounding structures. However, these results confirm our ability to capture a spectral fingerprint specific to the normal gland in deceased lambs, as well as our ability to distinguish it from various other surrounding tissues in new individuals. Previous studies using comparable Raman spectroscopy systems have achieved high classification accuracies (>90%) in distinguishing brain tumors from normal tissue during human surgical procedures.^32^ These promising findings suggest that pituitary adenoma classification should be further explored in a clinical study involving human patients.

For multi-class model II, the slightly lower accuracy (>95%) during cross-validation is likely due to the reduced dataset size that the model was trained on during fivefold cross-validation, combined with the similarity of the spectral fingerprints of sella turcica dura mater and bone, which were the only misclassified classes. However, the perfect classification of the model on the testing set comprising new individuals illustrates the high potential of the probe as a multi-class tissue classifier to guide safe transsphenoidal endonasal surgery, as it would allow real-time mapping of any tissue whose exact nature is unsure during the operation.

Regarding the comparison of the different acquisition methods, the differences in spectral variance and the results of the similarity measurements among the methods can be attributed to greater contamination of the pituitary gland signal by surrounding tissues in the *ex situ* method and even more so in the *in situ* method. Residual tissue from the cutting process used to expose the gland is likely more present on the surface in both *in situ* and *ex situ* spectra, despite rinsing the half-heads, compared with in-section gland spectra exposing the inner gland. In the *in situ* method, additional contamination may occur due to improper probe placement near tissue boundaries, causing the probe to detect scattered photons from adjacent tissues within the illuminated volume. Measurements performed on resected specimens seem to capture the Raman signal of the gland more accurately than those performed *in situ*. The results also highlight that differences among the acquisition methods seem to introduce more variability in the spectra than differences among individuals.

Considering all factors, a precise and accurate definition of the exact structures included in the illumination volume of the probe is crucial for a clinical study, whose first objective would be to acquire various spectral fingerprints characteristic of specific tissues. Accurate labeling of the spectra collected to train classification models is paramount. Measurements taken *in vivo* during surgery and their localization should be accurately correlated to resected specimens to obtain their true label. Although the impact of measurements taken with different acquisition methods should be monitored, *ex vivo* measurements on tissues after their resection could prove useful. These measurements could present less cross-contamination of the other structures and various liquids associated with surgeries. They could validate and provide insights into the *in vivo* acquisitions and increase the size of the dataset, a key factor in machine learning efficiency.

## Supplementary Material

10.1117/1.JBO.30.3.035004.s01

## Data Availability

The data and material information that support the findings of this study are available from the corresponding authors upon reasonable request.
