# Supplementary material for: Development and preclinical evaluation of an endonasal Raman spectroscopy probe for transsphenoidal pituitary adenoma surgery
Source: J Biomed Opt. 2025 Mar 20;30(3):035004. doi: 10.1117/1.JBO.30.3.035004 (PMC11924674; doi:10.1117/1.JBO.30.3.035004)
Supplement: Supplementary file 1 [file JBO_030_035004_SD001.docx]

**Supplementary Material**


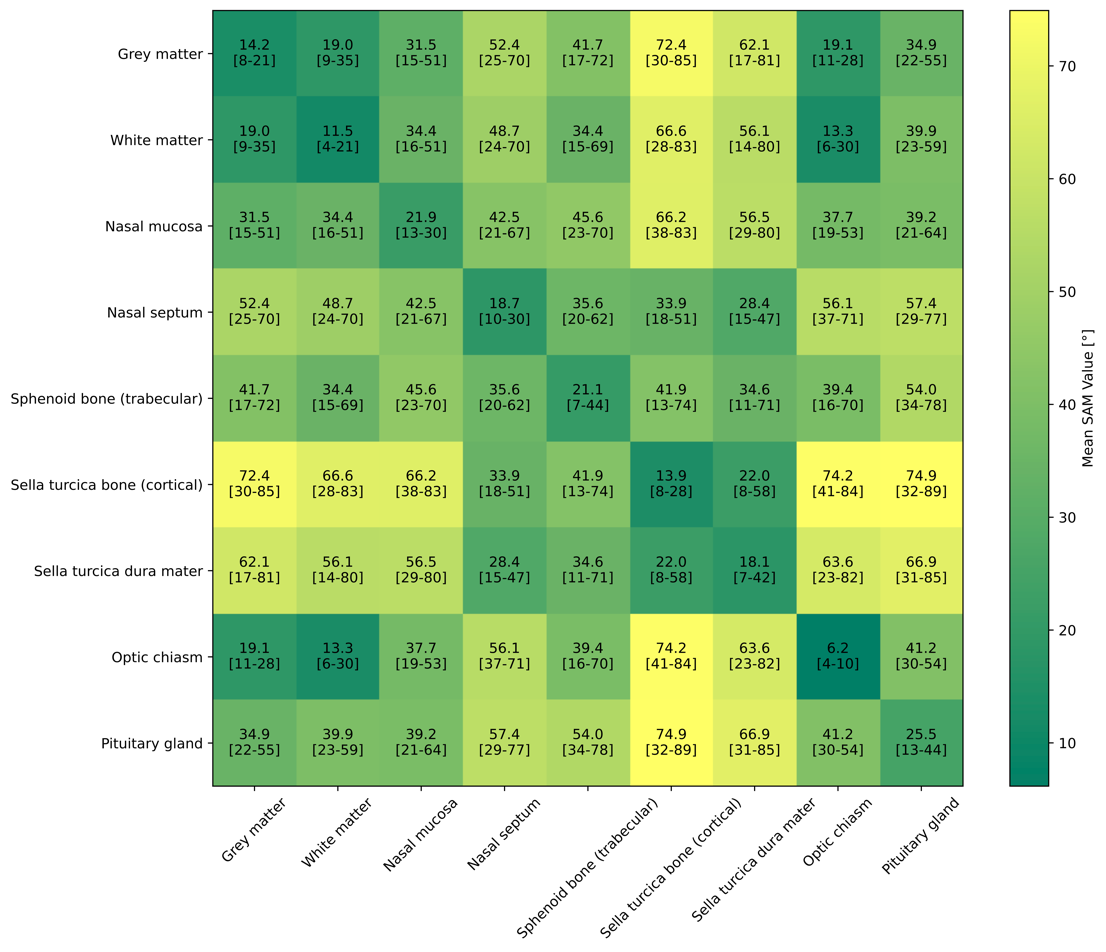


**Fig. S1** Spectral Angle Mapper (SAM) values computed between individual Raman spectra of tissue pairs. For each pair, the mean SAM value is shown, along with the range indicating the minimum and maximum observed angles.
